# An In Vivo Comparison: Novel Mesh Suture Versus Traditional Suture-Based Repair in a Rabbit Tendon Model

**DOI:** 10.1016/j.jhsg.2021.10.003

**Published:** 2021-11-19

**Authors:** Lindsay E. Janes, Lauren M. Mioton, Megan E. Fracol, Jason H. Ko

**Affiliations:** ∗ Division of Plastic and Reconstructive Surgery, Department of Surgery, Northwestern University Feinberg School of Medicine, Chicago, IL

**Keywords:** Biomechanical study, Mesh suture, Tendon repair

## Abstract

**Purpose:**

Despite advancements in surgical techniques, suture pull-though and rupture continue to limit the early range of motion and functional rehabilitation after flexor tendon repairs. The aim of this study was to evaluate a suturable mesh compared with a commonly used braided suture in an in vivo rabbit intrasynovial tendon model.

**Methods:**

Twenty-four New Zealand female rabbits (3–4 kg) were injected with 2 units/kg botulinum toxin evenly distributed into 4 sites in the left calf. After 1 week, the animals underwent surgical tenotomy of the flexor digitorum tendon and were randomized to repair with either 2-0 Duramesh suturable mesh or to 2-0 Fiberwire using a 2-strand modified Kessler and 6-0 polypropylene running epitendinous suture. Rabbits were killed at 2, 4, and 9 weeks after surgery.

**Results:**

Grouping across time points, 58.3% (7 of 12) of Duramesh repairs were found to be intact for the explant compared with 16.7% (2 of 12) of Fiberwire repairs (*P* = .09). At 2 weeks, the mean Duramesh repairs were significantly stronger than the Fiberwire repairs with a mean failure load of 50.7 ± 12.7 N compared to 14.8 ± 18.3 N (*P* = .02). The load supported by the Duramesh repairs at 2 weeks (mean 50.7 ± 12.7 N) was similar to the load supported by both Fiberwire (52.2 ± 13.6 N) and Duramesh (57.6 ± 22.3 N) at 4 weeks. The strength of repair between Fiberwire and Duramesh at 4 weeks and 9 weeks was not significantly different.

**Conclusions:**

The 2-strand tendon repair with suturable mesh achieved significantly greater strength at 2 weeks than the conventional suture material. Future studies should evaluate the strength of repair prior to 2 weeks to determine the strength curve for this novel suture material.

**Clinical Relevance:**

This study evaluates the utility of a novel suturable mesh for flexor tendon repair in an in vivo rabbit model compared with conventional suture material.

Successful tendon repair is vital for restoration of hand function after trauma, but it can be plagued with complications, including adhesion formation, joint stiffness, and repair rupture. As such, numerous studies analyzing repair techniques have focused on the initial fixation strength, gap formation, and repair failure in an attempt to define the ideal tendon repair and rehabilitation protocol. In the setting of flexor tendon injuries, studies have shown that early motion and multistrand core repairs are beneficial in long-term outcomes.^1^, ^2^, ^3^, ^4^, ^5^, ^6^ Despite continued advancements in suture techniques and materials, complications still occur in nearly 15% of cases, with repair rupture occurring in almost 5% of all cases.^7^^,^^8^ These complications impact patient quality of life, leading to additional surgery and prolonged or permanent functional deficits.^9^^,^^10^ Therefore, continued investigation is needed to improve tendon repair strength and longevity to decrease repair failure under physiologic conditions.

A novel suturable mesh (Duramesh suturable mesh, Fig. 1) has recently been used for the repair of abdominal wall defects with notable decreases in suture pull-through and subsequent hernia formation.^11^, ^12^, ^13^ The success of this suturable mesh is thought to be due to its larger surface area and macroporous design that permits fibrovascular ingrowth and encapsulation of individual filaments.^14^^,^^15^ Made of 12 polypropylene filaments woven into an open cylindrical crosshatch configuration, the structural design of the suturable mesh used in this protocol has been shown to resist suture pull-through.^16^ As with abdominal wall repairs, flexor tendon repairs represent a separation of soft tissues that are subject to high, dynamic tensile forces that can result in repair failure with standard suture material. In a cadaver model of flexor tendon repairs with a 4-strand core cruciate suture configuration, the suturable mesh was found to endure a greater number of cycles and higher loads before failure compared to conventional suture.^17^ In a cadaver model of tendon coaptation constructs using a single pass side-to-side coaptation, the suturable mesh was equal in strength and bulk to the conventional suture, although it had a higher gliding resistance of the construct.^18^

**Figure 1:**
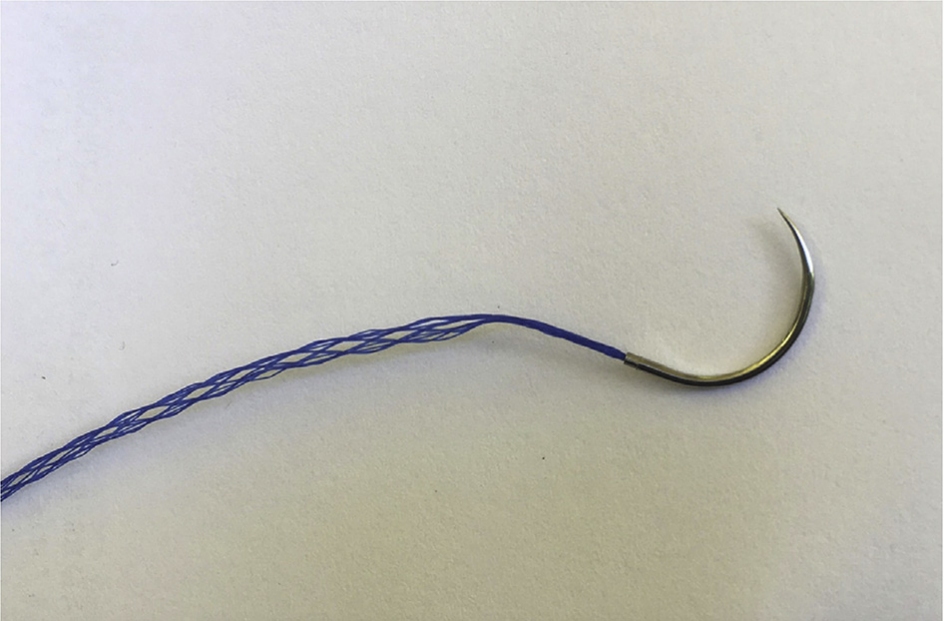
Duramesh suturable mesh.

Fibrovascular ingrowth between and around the filaments could potentially improve overall repair strength and would add to the resistance to the pull-through demonstrated in cadaveric models. The aim of this study was to evaluate the efficacy of a novel suturable mesh for tendon repair in an in vivo model. We hypothesized that the suturable mesh, as measured by the load at repair failure, will have improved biomechanical properties compared with the 2-0 Fiberwire suture.

## Materials and Methods

### Animal model

Surgical procedures and perioperative care measures were conducted in compliance with the Northwestern University Institutional Animal Care and Use Committee and the National Institutes of Health guidelines. Establishment of the final protocol for successful tenotomy and tendon repair in the hindlimbs of New Zealand female rabbits weighing 3–4 kg required several protocol modifications as detailed below, each approved by the Northwestern University Institutional Animal Care and Use Committee.

**Figure 2:**
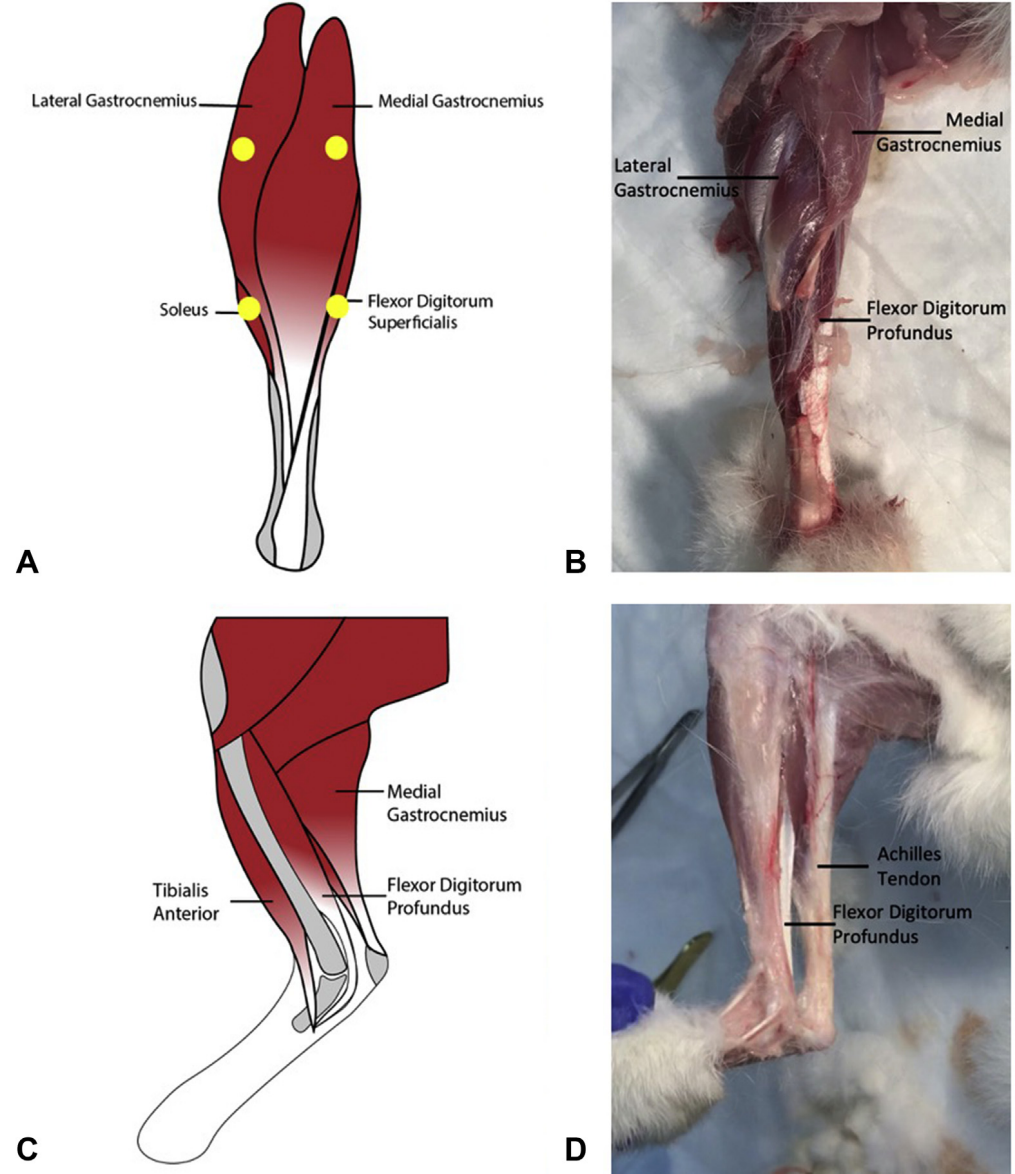
**A** and **B** demonstrate a posterior view of the rabbit hindlimb, demonstrating the approximate location of the 4 injection sites (yellow circles). Additionallynote how the Achilles tendon components are not well coalesced until just prior to the bone insertion. **C** and **D** demonstrate the location of the flexor digitorum profundus tendon deep to the Achilles tendon.

The pilot studies had minimal to no success in achieving any intact tendon repairs. Initially, we used an extrasynovial Achilles tendon model, and after tenotomy and repair, the rabbits were casted to immobilize the repair for 1 week. However, the rabbits did not tolerate the casts, and all repairs were ruptured with the force that was generated to kick off the cast. Second, we evaluated tenotomy and repair of half of the Achilles tendon so that the remaining half would internally stabilize the repair. However, the tendinous portions of the lateral gastrocnemius, medial gastrocnemius, and flexor digitorum superficialis that comprise the Achilles tendon do not coalesce until just proximal to the bone (Fig. 2A, B) and thereby could not adequately stabilize the cut half. Thus, all of the repair attempts failed. Third, abandoning the Achilles tendon model, we tested tenotomy and repair of the deeper flexor digitorum tendon, which had the added benefit of being an intrasynovial tendon (Fig. 2C, D). Various suture techniques (MGH Becker, figure-of-8 and, modified Kessler) were used, and some of these were found to be successful but were not reliable without immobilization because of the high forces generated in the mobile rabbit hindlimb. Fourth, we evaluated botulinum toxin injected at 4 Botox units/kg into the gastrocnemius and flexor digitorum as guided by Tuzuner et al.^19^ However, the rabbits experienced signs of systemic botulism and had to be killed within 48 hours of injection.

**Figure 3:**
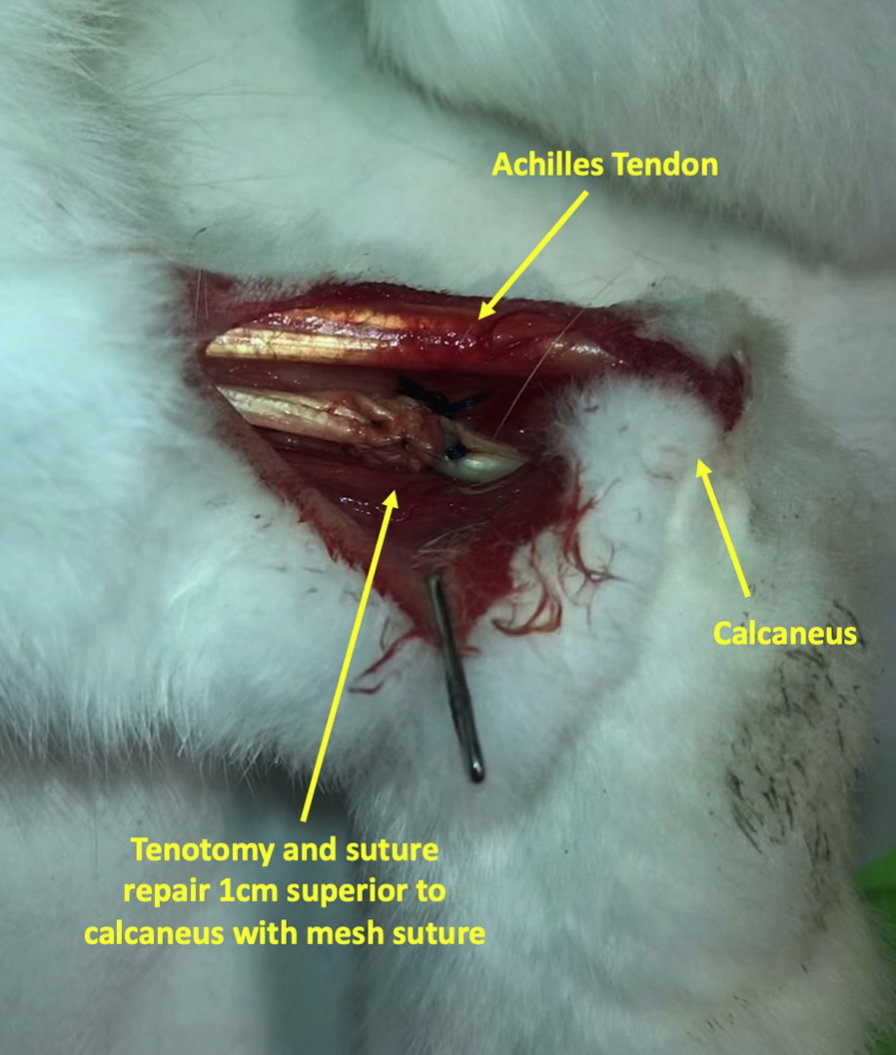
Tenotomy and suture repair of the flexor digitorum tendon approximately 1 cm superior to the calcaneous.

Finally, a successful model involving injection of 2 Botox units/kg into the gastrocnemius and flexor digitorum muscles 1 week prior to repair was established. The Botox was diluted to 50 units in 2.5 mL, and the dose was split evenly between 4 injection sites in the posterior hind limb muscle belly (Fig. 2A) determined by palpation of the posterior rabbit calf. After 1 week, we performed tenotomy of the flexor digitorum profundus tendon approximately 1 cm proximal to the calcaneus and repair using a modified Kessler repair core stitch with either 2-0 Duramesh or 2-0 Fiberwire, followed by a 6-0 polypropylene running epitendinous repair (Fig. 3). Three milliliters of 1% lidocaine with epinephrine were injected subcutaneously around the marked incision site prior to incision for pain control and hemostasis. A dose of meloxicam (0.2 mg/kg) was administered subcutaneously prior to the incision, and buprenorphine sustained release was administered subcutaneously for analgesia immediately after the procedure prior to emergence from anesthesia. In the final protocol, no orthosis or postoperative protocol was used to limit the motion. Although the injected limb was not completely paralyzed, the rabbits were unable to flex the ankle and toe joints to generate their typical hop. Instead, all movements from the limb were generated from the hip and knee joints. (Video 1, available on the *Journal’s* Web site at www.jhsgo.org) The gait changes because of the Botox injection remained present throughout all time points. This Botox protocol and use of the flexor digitorum profundus tendon represented a novel, reliable in vivo model to test the intrasynovial flexor tendon repair without immobilization, throughout this study.

### Tendon harvest and grading of tendon repair

Twenty-five animals were included in the final experiments comparing the 2-0 Duramesh with the 2-0 Fiberwire modified Kessler 2-strand core repairs; 24 were included for biomechanical testing, and 1 was evaluated for histology. In the in vivo rabbit tendon model, a 4-strand repair with 2-0 suturable mesh was too bulky, and thus a 2-strand modified Kessler was used. We elected to perform a 2-strand repair with conventional suture as well, as the goal of this study was to compare the sutures with a consistent technique. Animals were killed in accordance with the Institutional Animal Care and Use Committee procedures at 2 weeks, 4 weeks, and 9 weeks after the repair procedure. Each tendon was evaluated in situ and measured to determine if the tendon was intact; had 1 to 4 mm of gapping; or had ≥5 mm of gapping. The tendon was then removed, wrapped in a saline-soaked gauze, placed in a 50-mL plastic conical centrifuge tube, and immediately frozen at −4 °C. If there was gapping of the repair, the tendon ends were harvested along with the synovial sheath between them so that biomechanical testing of the construct could still be performed. No animals were excluded from the final analysis.

### Biomechanical testing

**Figure 4:**
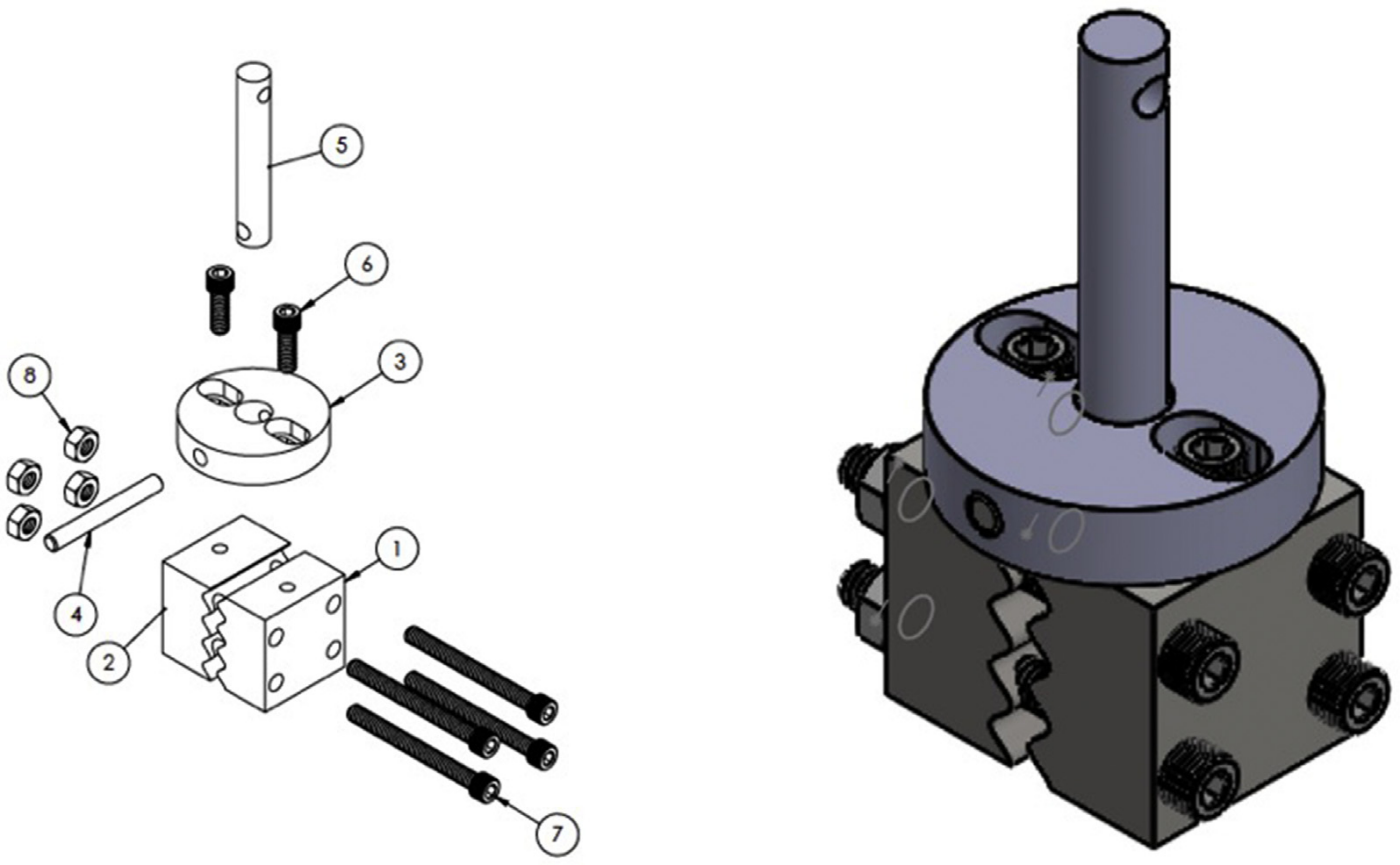
Custom manufactured cryoclamps for biomechanical testing. **1** Piece one of clamp with 4mm peak to peak distance of sine wave. Qty 1. **2** Piece two of clamp with inverse sine wave. Qty 1. **3** Base of attachment to tensometer. Qty 1. **4** Doyle to lock part 3 into part 5 quickly while clamp frozen. Qty 1. **5** Top of attachment to tensometer. Length 12 cm to keep liquid nitrogen off of machine. Qty 1. **6** Socket head cap screw. 92196A540 Qty 2. **7** Socket head cap screw. 92196A821 Qty 4. **8** Hex Nut. 91845A029 Qty 4.

Biomechanical testing of all samples was performed by a single researcher over 2 days. Because of the visible differences in the suture material upon tendon explant, blinding for the testing was not possible. Specimens were thawed and tested in random order. On the day of testing, the sample was removed from the freezer and placed in a warm water bath for 30 minutes to completely thaw the specimen. The specimen was then loaded into our custom manufactured cryoclamps (Fig. 4), designed as a modification of the method used by Bowser et al.^20^ The clamps were tested with intact rabbit tendon specimens and were found to maintain contact with the clamp without slippage until tendon failure at around 450 N. After loading the specimen into the clamp, the body of the clamp was dipped in liquid nitrogen for 1 minute, ensuring that the sample was not submerged in liquid nitrogen. After 1 minute, the clamps were removed and placed on the countertop. If the specimen was partially frozen due to the proximity of the liquid nitrogen, it was warmed between 2 fingers until it was no longer cold to the touch. The clamps were then mounted on the Instron Test System, Model 5942 (Instron). Specimens were preloaded to 1 N and cycled for 5 cycles of 0 to 25 N at 1 N/s, followed by a load to failure at 1 N/ s. Precycling to 25 N was chosen based on an in vivo study by Schuind et al^21^ that measured flexor tendon forces in patients undergoing carpal tunnel surgery. The study determined that a mean force of 19 N was required to produce active distal interphalangeal flexion and 25 N to produce active thumb flexion.

Loading protocols used in biomechanical studies examining the strength of flexor tendon repair at gapping and failure are widely variable.^22^ Many studies use the load to failure alone, whereas others use cyclic preloading followed by load to failure or cyclic loading to failure.^3^^,^^23^, ^24^, ^25^ Cyclic loading is thought to more accurately reflect the physiologic forces placed on tendon repair during therapy or during exercise. Additionally, it was determined that the cyclic testing demonstrates a gap formation at lower loads than does static load to failure testing.^26^ However, because of the time constraints set by the cryoclamps, which most effectively adhere the specimen to the machine for accurate measurements, we elected to perform the combined protocol described above that took an average of 194.1 ± 83.5 seconds (maximum, 343.5 seconds).

### Histology

Two in vivo tendon repair samples, 1 with Duramesh and 1 with Fiberwire, which were not included in the biomechanical testing, were harvested at 2 weeks after the repair procedure. The samples were placed in formalin for 48 hours and then dehydrated overnight. The following day, the samples were embedded in paraffin. Sections that were 10-μm thick were cut and mounted on slides. These were then stained using a standard hematoxylin-eosin staining protocol. Images of tissue sections were taken on a Nikon Eclipse 50i microscope and tissue ingrowth was rated according to the American Society for Testing and Materials (ASTM) ingrowth scale.^27^

### Statistics

The sample size was determined using previous biomechanical data comparing suturable mesh and Fiberwire tendon repairs in human cadaver tendons.^17^ The average load required to produce 1 mm of gapping in 3-0 Fiberwire was 42 ± 7 N compared with 58 ± 7 N in suturable mesh.^17^ A minimum sample size of 6 tendons per time point (3 per suture type) was estimated using an α value of 0.05 and power of 0.80, to be able to detect a significant difference in the load required to generate a 1 mm gap. The analysis of the in situ repair grade at the time of harvest was performed by grouping all time points and using the Fisher exact test. The analysis of biomechanical testing was performed with Welch *t* test comparing values at each time point. Confidence interval was 95%, and statistical significance was defined with a *P* value of <.05.

## Results

### Tendon repair grading

**Figure 5:**
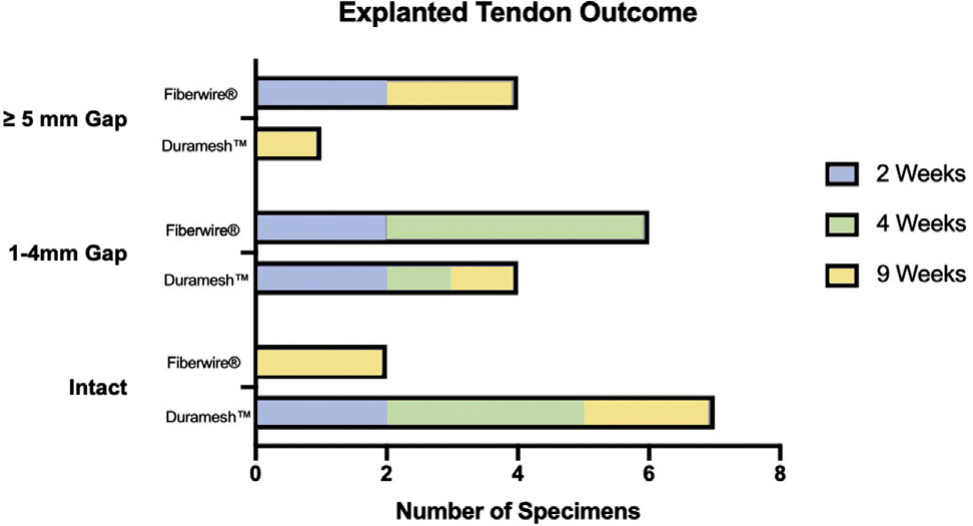
In vivo grade of tendon repair at explant.

**Figure 6:**
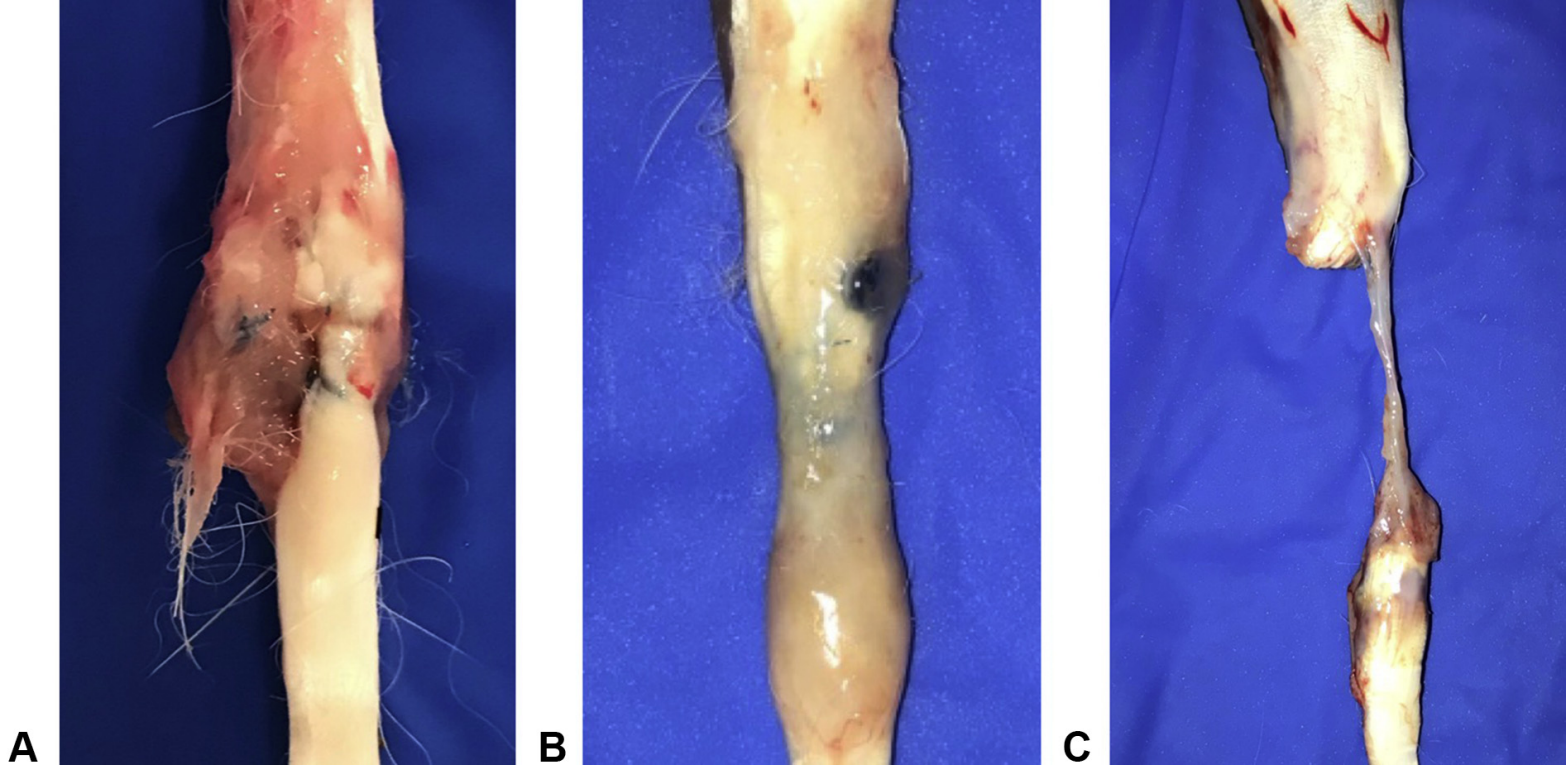
Examples of tendon repair grading at explant. **A** Intact at 2 weeks (Duramesh suturable mesh). **B** 1–4-mm gap at 4 weeks (Duramesh suturable mesh). C >5-mm gap at 2 weeks (Fiberwire suture).

Grouping across time points, 58.3% (7 of 12) of Duramesh repairs were found to be intact at explant compared with 16.7% (2 of 12) of Fiberwire repairs (*P* = .09), and 33.3% (4 of 12) of Fiberwire repairs had greater than 5-mm gapping compared with only 8.3% (1 of 12) of Duramesh repairs (Fig. 5). Figure 6 provides examples of tendon repair grading at explantation.

### Biomechanical testing

**Figure 7:**
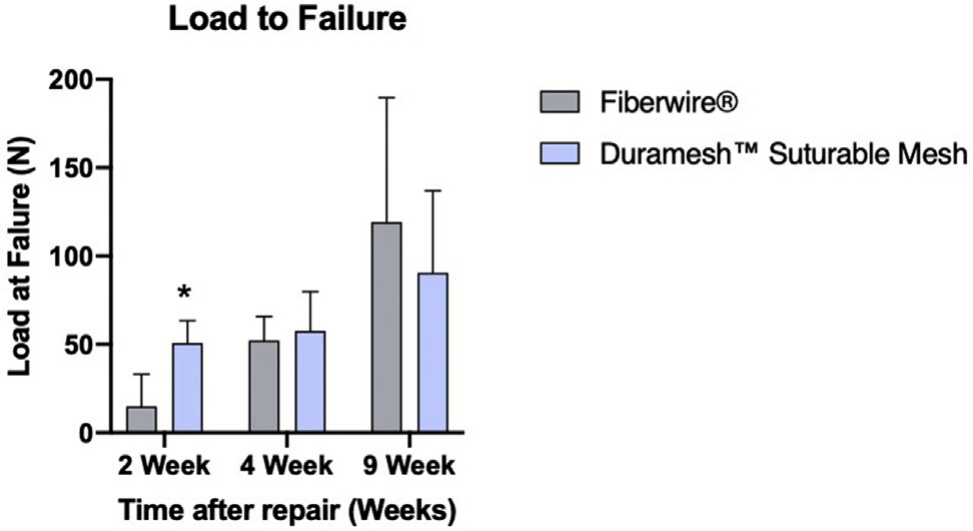
Load to failure at study time points evaluated. Tendons were precycled with 5 cycles of 25 N at 1 N/s, followed by load to failure at 1 N/s. ∗Statistical significance, *P* < .05.

At 2 weeks, the Duramesh repairs were found to be significantly stronger than the Fiberwire repairs (*P* = .02). The load supported by the Duramesh repairs at 2 weeks (mean 50.7 ± 12.7 N) was similar to the load supported by both Fiberwire (52.2 ± 13.6 N) and Duramesh (57.6 ± 22.3 N) at 4 weeks. The strength of repair between Fiberwire and Duramesh at 4 weeks and 9 weeks was not significantly different (Fig. 7 and Table 1). A sample video of the biomechanical testing is featured in Video 2 (available on the *Journal’s* Web site at www.jhsgo.org).

**Table 1:** Mean Strength of the Biomechanical Load at Failure (in Newtons)

Study Time Point	2-0 Fiberwire Suture	2-0 Duramesh Suturable Mesh	*P* value
2 weeks	14.8 ± 18.3	50.7 ± 12.7	.021∗
4 weeks	52.2 ± 13.6	57.6 ± 22.3	.697
9 weeks	119.2 ± 70.4	90.5 ± 46.4	.525

∗ Statistical significance, *P* < .05

### Histology

**Figure 8:**
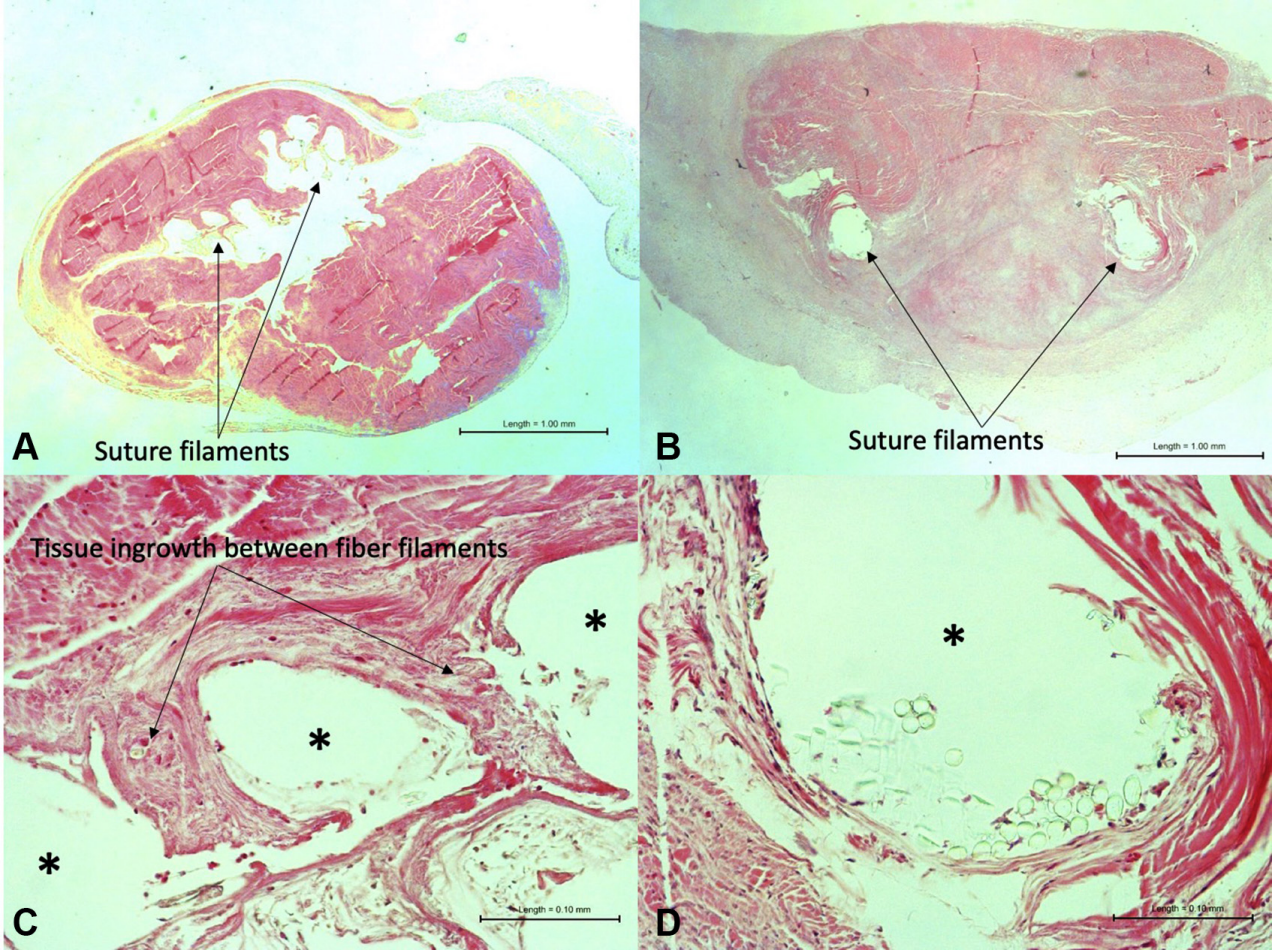
Histology images of tendon repairs harvested 2 weeks after repair demonstrate tissue ingrowth into the Duramesh construct but not the Fiberwire construct. **A** Duramesh tendon repair. **B** Fiberwire tendon repair. **C** Duramesh tendon repair, with asterisk indicating the locations of the fiber filaments. Collagen deposition between filaments is indicated by the arrows. **D** Fiberwire tendon repair, with asterisk indicating the location of the fiber (**A** and **B** magnification × 2; **C** and **D** magnification × 20).

The histology images for tendon repairs harvested at 2 weeks are shown in Figure 8. Tissue ingrowth between the polypropylene filaments was visible on the Duramesh repair (ASTM grade 2, moderately thick bands of fibroblasts and collagen deposits between mesh filaments). There was no tissue ingrowth into the Fiberwire suture site (ASTM grade 0, none). Table 2 shows the ASTM grading scale.^27^

**Table 2:** American Society for Testing and Materials Tissue Ingrowth Scale^27^

Score	0	1	2	3
Tissue ingrowth	None	Fair: thin bands of fibroblasts and small collagen deposits between mesh filaments	Good: moderately thick bands of fibroblasts and collagen deposits between mesh filaments	Excellent: all spaces between mesh filaments occupied by fibroblasts, collagen deposits, and capillaries

## Discussion

Successful flexor tendon repair and rehabilitation are a function of the strength of the suture-tendon construct and the time that it takes to achieve that strength before significant adhesions can form. Early active motion protocols reduce peritendinous adhesion formation and joint stiffness; however, they risk gap formation and repair failure.^7^^,^^10^^,^^28^ Elucidating the ideal suture material, suture size, and repair pattern that can withstand early mobilization has been the basis for the majority of tendon biomechanical research over the past 20 years. However, all these studies have focused on refining the repair techniques with small diameter sutures that are susceptible to suture pull-through and failure. We present a reliable in vivo rabbit model of intrasynovial flexor tendon repair and data comparing the suturable mesh with the conventional small diameter suture material.

Small diameter sutures concentrate force and pressure at the suture-tissue interface, leading to acute or chronic suture pull-through, gap formation, and surgical failure. Increasing the number of core strands has been shown to increase the load a tendon repair can tolerate before gap formation and repair failure.^29^^,^^30^ Analogous to laparotomy closures, distributing the total load of the suture-tissue interface across more core strands (greater surface area), decreases the force experienced at each site, thus decreasing suture pull-through.^31^ Simple calculations using the surface area of a cylinder results in a 57-mm^2^ surface area for each 1-cm length of Duramesh, as opposed to a 10-mm^2^ surface area for a 2-0 polypropylene suture with a filament diameter of 0.32 mm. The 12 filaments of suturable mesh for the 2-0 size are each the size of a 5-0 polypropylene suture, and when they come together, they can fit into the end of a standard swaged needle. The increased suturable mesh surface area inherently increases the size of the suture-tissue interface, which lowers the acute force at the interface, potentially reducing tearing for a single core strand. In this study, across the time points evaluated, 58.3% (7 of 12) of Duramesh repairs were found to be intact at explant compared with 16.7% (2 of 12) of Fiberwire repairs, although this was not statistically significant. We attributed this to the decreased suture pull-through within the first few hours to days following the repair.

In addition to the increase in the total surface area at the suture-tissue interface, the strength of any repair is the sum of the physical construct (suture) and the scar of biologic healing. The foreign body response to encapsulate or integrate is dependent on the porosity of the material, the polymer composition, and the filament size.^15^ Prior histologic evaluation of a suturable mesh prototype used in rat hernia repair demonstrated that there was excellent fibrovascular ingrowth of the surrounding tissue into the macroporous structure.^12^ With time, each individual filament undergoes a microencapsulation, thereby functioning as a scar scaffold. We expect that this tissue ingrowth, as observed histologically in Figure 8, is what contributes to the greater strength of repair at 2 weeks in this study compared to conventional suture. The greater strength of repair for the Duramesh suture than the Fiberwire at 2 weeks presents a significant advantage in the allowance of early active motion protocols.

Future directions for this research using this intrasynovial flexor tendon model include creating a strength curve for the suturable mesh from the time of tendon repair to the 2-week time point so that the ideal time to initiate early active motion can be elucidated. In a porcine model, laparotomy closures using a suturable mesh prototype had steeper and longer force-elongation curves than that of 0-polypropylene ones, as early as 8 days after the operation.^11^ Additionally, at the time of explantation, it was noted that the scar tissue surrounding the repair was greater in the suturable mesh repairs. Thus, although the increased scar formation promoted by the suturable mesh within the tendon construct may increase the strength of repair, it may also increase adhesion formation. Recent work by Gillis et al^18^ reported an increased gliding resistance in tendon coaptations performed with suturable mesh and conventional suture, and further examination of this is warranted. Currently, the 2-0 suturable mesh is the smallest size available for research. When this suture gets cleared for sale, larger suture sizes with additional numbers of filaments will be available, and so this in vivo tendon research can be further pursued using larger (but not smaller) animal models.

One limitation of this study is the unknown natural time course of healing of rabbit tendons compared with that of human tendons. With the exception of a single study in the rabbit forepaw, 2 in chickens, and 1 in a turkey, the majority of in vivo tendon healing studies have been performed in canine models.^32^, ^33^, ^34^, ^35^, ^36^, ^37^, ^38^, ^39^, ^40^ However, canine studies are expensive and encounter significant regulatory limitations. We believe that this rabbit hindlimb model presents a new and exciting alternative model for future studies of tendon healing, although the correlation between rabbit and human tendon healing must be further studied. An additional limitation of this study is the 2-strand tendon repair used in this model rather than a minimum 4-strand repair that is used clinically. The rabbit flexor digitorum tendon is slightly smaller in caliber than the human flexor tendon, and currently, the 2-0 suturable mesh is the smallest size available for research. The 4-strand repairs with the 2-0 suturable mesh were too bulky and damaging to the tissue to be used in this study. Further studies with smaller suture sizes and an increased number of core strands for repair should be conducted in the future.

In this study, we have used a novel intrasynovial flexor tendon repair model to demonstrate that the tendon repair with suturable mesh achieved significantly greater strength at 2 weeks than the conventional suture material. Future studies should evaluate the strength of repair prior to 2 weeks to determine a strength curve for this novel suture and whether suturable mesh repair may allow for early, possibly immediate, motion protocols after flexor tendon repair.

## Figures and Tables

**Video 1 Still:**
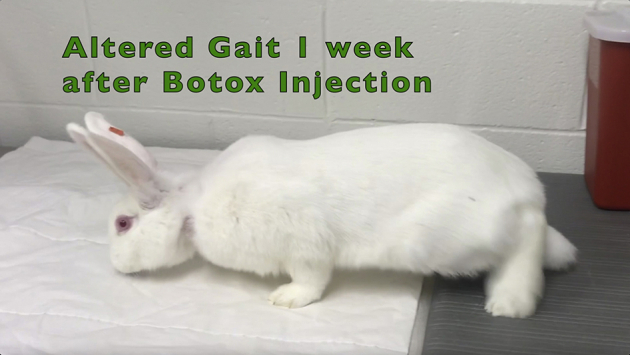


**Video 2 Still:**
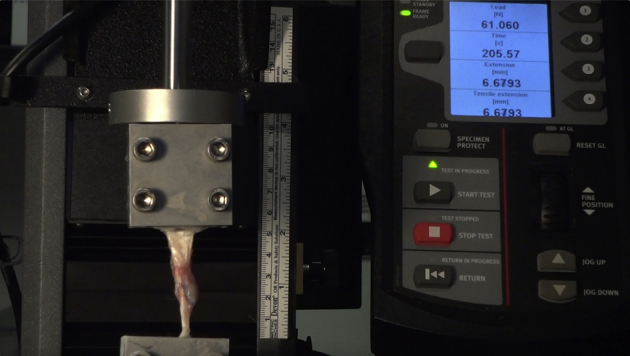


## Supplementary Data

Video 1 Demonstration of rabbit’s altered gait after immobilization of ankle and toe flexors with botulinum toxin.

Video 2 Biomechanical testing set up with Duramesh Suturable Mesh repair explanted at two weeks during its load to failure phase of biomechanical testing.
